# Our Advertising Department

**Published:** 1876-07

**Authors:** 


### Editorial and Miscellaneous.

---

This journal is on file with Dr. C. W. Bernacki, 344 West 30th street, New York City, Medical Journal Advertising Bureau, where advertising contracts can be made.

*Our Advertising Department.*—We respectfully urge our readers to examine carefully our advertisements in every issue, as we are diligently seeking to bring before them notices of things practical and interesting, and to present them first class houses only, such as they may feel safe in recommending to their home druggists.

The colleges which we advertise will also be first class, and we will in no case allow, knowingly, a bogus concern the use of our pages for any purposes. Preceptors may thus learn what schools to recommend to their pupils; for, after all, much of the responsibility for the support of these irregular schools and the consequent low standard of education among the graduates is due to the preceptors throughout the country. This very point was made at the convention of editors by a college professor, who argued that it was the duty of physicians everywhere to take such students only into their offices as were possessed of satisfactory preliminary educations; and we here add that it is not less their duty to recommend them to first class colleges.

